# Immunotherapy for Thymomas and Thymic Carcinomas: Current Status and Future Directions

**DOI:** 10.3390/cancers16071369

**Published:** 2024-03-30

**Authors:** Arun Rajan, Alisa K. Sivapiromrat, Meredith J. McAdams

**Affiliations:** Thoracic and Gastrointestinal Malignancies Branch, Center for Cancer Research, National Cancer Institute, National Institutes of Health, Bethesda, MD 20892, USA

**Keywords:** thymoma, thymic carcinoma, immunotherapy, immune checkpoint inhibitors

## Abstract

**Simple Summary:**

Immune checkpoint inhibitors have revolutionized cancer therapy and improved clinical outcomes. Immunotherapy is now increasingly combined with chemotherapy and other conventional treatments, such as radiation therapy, as part of the multimodal treatment of earlier-stage cancers. Although generally well tolerated and capable of inducing long-lasting responses, immunotherapy for thymic epithelial tumors can be challenging due to defects in immune self-tolerance, which increase the risk of immune-mediated toxicity. In order to improve the safety of immunotherapy and maximize clinical benefit in patients with thymic cancers, there is a pressing need to identify potential biomarkers of response and toxicity for this patient population. In this paper, we review the current role of immunotherapy for thymic cancers and discuss future applications across the spectrum of stage and histology of these diseases.

**Abstract:**

Thymic epithelial tumors are a histologically diverse group of cancers arising from the epithelial compartment of the thymus. These tumors are characterized by a low tumor mutation burden, a lack of actionable genomic changes, and, especially with thymomas, defects in immune tolerance. Surgery is the mainstay of the management of resectable disease, whereas advanced, unresectable tumors are treated with platinum-based chemotherapy. Disease recurrence can occur months to years after frontline treatment. Although several options are available for conventional treatment of recurrent thymic tumors, response rates are generally low, and treatment-related toxicity can affect quality of life. A subset of patients benefit from biologic therapies, but there remains an unmet need for the development of new treatments. Immune checkpoint inhibitors are safe, clinically active, and have contributed to an improvement in survival for patients with a wide variety of cancers. However, the application of these revolutionary treatments for thymic cancers is limited to their use for the management of recurrent thymic carcinoma because of the risk of immune toxicity. In this paper, we review the current uses of immunotherapy for the management of thymic epithelial tumors and highlight potential strategies to improve safety and broaden the application of these treatments for patients with thymic cancers.

## 1. Introduction

Thymomas, thymic carcinomas, and thymic neuroendocrine tumors, collectively referred to as thymic epithelial tumors (TETs), are rare cancers with an annual incidence of 0.48 (for thymic carcinomas) to 2.2 (for thymomas) per million individuals in the United States [1]. TETs arise from the epithelial compartment of the thymus and are classified into distinct histological subgroups by the World Health Organization (WHO) based on the morphology of epithelial cells, retention of organotypic features, presence of intermingled immature T cells, and presence of nuclear atypia and pleomorphism [2]. Molecular features include a low tumor mutation burden and a lack of currently actionable genomic aberrations [3,4]. One of the most unique features of TETs is the predisposition to paraneoplastic autoimmunity due to a breakdown in thymic tolerance, which leads to the persistence of autoreactive T cells [5]. Consequently, patients with TETs, predominantly thymoma, can develop a variety of autoimmune and immunodeficiency disorders [6].

The clinical behavior of TETs can vary from relatively indolent to highly aggressive. The histologic subtype of the tumor, stage of disease, tumor size, and completeness of surgical resection are important prognostic factors [7]. Ten-year overall survival (OS) can range from greater than 80% for patients with early-stage disease to less than 40% for patients with advanced or unresectable disease [8]. Surgical resection represents the cornerstone of the management of TETs, where applicable. For patients presenting with unresectable disease at diagnosis, induction chemotherapy can render the disease resectable in a subset of patients [8]. Post-operative radiation therapy is associated with a survival benefit in patients with locally advanced disease and in individuals with microscopic residual disease after surgery [8,9]. Frontline treatment of advanced, unresectable TETs consists of definitive platinum-based chemotherapy, with several trials reporting response rates of 30% to 60% based on the chemotherapy combination and the histology of the tumor [10]. Disease relapse occurs often in patients with advanced TETs, and options for further systemic therapy are limited and generally characterized by low response rates and a modest impact on survival [11]. These observations call for the development of newer treatments for TETs. However, the paucity of actionable genomic aberrations and the identification of a relatively limited number of other novel targets have been obstacles to drug development for TETs. It is against this backdrop that ongoing research efforts have focused on the development of immunotherapy for TETs.

Immunotherapy is a broad term that encompasses a variety of interventions designed to initiate or amplify an adaptive immune response against a tumor by targeting tumor-associated antigens (TAAs) or neoantigens, modulating immune checkpoints, or using modified immune cells such as chimeric antigen receptor (CAR) T cells [12,13]. Immune checkpoint inhibitors (ICIs) have revolutionized the treatment of several cancers and demonstrated the ability to elicit durable responses and improve survival [14]. In recent years, there has been a renewed focus on developing novel combinations of ICIs with other forms of immunotherapy and other conventional therapies and the incorporation of immunotherapy into treatment regimens for patients with early-stage and locally advanced cancers to further improve clinical outcomes [15,16]. In spite of these promising developments, the adoption of ICIs and other forms of immunotherapy for the management of TETs has been hampered by the substantial risk of immune-mediated toxicity arising from defective immune tolerance associated with these tumors. In this review, we describe the current role of ICIs for the management of TETs and outline efforts to broaden the use of immunotherapy for these diseases by developing strategies to improve safety and identify newer targets for immunomodulation.

## 2. Biological Considerations for Use of Immunotherapy

Identification of the predictors of the clinical activity and safety of immunotherapy is an active area of research, especially in relation to ICIs [17,18,19,20]. Potential biomarkers of response and resistance to ICIs can be broadly divided into host- and tumor-specific factors. Among these features, tumor cell expression of programmed death-ligand 1 (PD-L1), tumor mutation burden (TMB), presence of mismatch repair deficiency (dMMR), or high microsatellite instability (MSI-H) have found broad clinical application and are used to identify patients likely to benefit from ICIs [18].

TETs exhibit relatively high degrees of PD-L1 expression, with frequencies of 23–92% in thymomas and 36–100% in thymic carcinomas, an observation that provided a rationale for the investigation of immune checkpoint blockade in recurrent TETs [21]. However, the utility of PD-L1 expression as a predictive biomarker for ICI therapy of advanced or recurrent TETs has been called into question due to the high frequency of PD-L1 expression in non-neoplastic thymus and the potential effect of prior systemic therapies on PD-L1 expression in recurrent TETs [22]. Among other well-recognized markers of response to ICIs, TETs have a low TMB and rarely exhibit MSI, which has been documented in 0.3% of thymomas and 2.3% of thymic carcinomas [3,4]. Emerging data suggest that autoimmune regulator (AIRE) deficiency, frequently observed in thymomas [23], increases sensitivity to immune checkpoint blockade [24]. These observations highlight the role of less well-recognized features of TET biology that have the potential to increase responsiveness to immune checkpoint blockade and could provide an explanation for the activity of ICIs observed in clinical trials.

Additionally, TETs have been shown to express a variety of proteins at high frequencies that function as TAAs and can be utilized for the development of novel forms of immunotherapy. A few examples include Wilms Tumor-1 (WT1), mesothelin, and cancer testis antigens (CTAs).

The Wilms Tumor-1 (WT1) protein is encoded by the *WT1* gene and has been shown to have an oncogenic role in several cancers [25]. In a small series of 18 cases, WT1 protein overexpression was detected in 80% of thymomas and 85% of thymic carcinomas [26]. Several recent trials have evaluated cancer vaccines directed against WT1, including a phase II trial for advanced TETs [26,27].

Mesothelin is a cell-surface glycoprotein with high frequencies of expression in several cancers, including up to 79% of thymic carcinomas, but with limited expression in normal tissues [28]. Immunotherapeutic strategies under development to target mesothelin include the use of immunotoxins, chimeric monoclonal antibodies, antibody–drug conjugates, cancer vaccines, and CAR T cells [29].

CTAs are a group of immunogenic TAAs that are attractive candidates for cancer immunotherapy due to their limited physiologic expression [30]. TETs show variable expression of the CTAs MAGE-A, NY-ESO-1, MAGE-C1, SAGE, and GAGE7, with higher frequencies of expression generally detected in thymic squamous cell carcinomas [31]. These observations support the development of CTA-directed immunotherapy for TETs.

TETs have a predisposition toward autoimmunity and immune-mediated toxicity that is deeply rooted in thymic biology and has limited the development of immunotherapy for these tumors. Thymic epithelial cells (TECs) are central to the evolution of lymphoid progenitors into immunologically competent T cells [32]. T cell development involves a complex series of coordinated steps controlled by various cytokines and chemokines during the passage of lymphoid progenitors from the thymic cortex to the thymic medulla [32]. Medullary TECs, under the control of the transcription factors AIRE and forebrain-expressed zinc finger 2 (Fezf2), express tissue self-antigens (TSAs), and developing T cells reactive to these TSAs undergo deletion by the process of negative selection, whereas immunologically tolerant regulatory T cells (Tregs) undergo expansion and generate immunological tolerance [5,32,33]. In addition to decreased expression of AIRE, other factors that contribute to the lack of thymic tolerance in TETs include altered thymic architecture, decreased expression of human leucocyte antigen class II, the inability to generate FoxP3+ Tregs, and genetic polymorphisms that affect T cell signaling [5,6,33].

A broad range of autoimmune disorders have been documented in patients with TETs, especially those with type B thymomas [6]. Less well-recognized manifestations of defective thymic tolerance include acquired immunodeficiency due to the presence of anti-cytokine autoantibodies and organ-specific conditions such as lymphocyte-driven autoimmune pneumonitis and a novel autoimmune endocrinopathy driven by an autoimmune response to pituitary-specific transcription factor-1 (PIT-1) [34,35,36]. Intriguingly, despite an overall increase in the prevalence of autoimmunity in patients with thymoma, the frequency of myasthenia gravis (MG) is disproportionately high in comparison with other conditions associated with AIRE deficiency, such as autoimmune polyendocrinopathy candidiasis ectodermal dystrophy (APECED) [37]. One possible mechanism for the high prevalence of neuromuscular autoimmunity in patients with thymoma might involve the autoimmunization of nascent T helper cells against acetylcholine receptor (AChR) epitopes that eventually results in the development of AChR autoantibodies, which target the neuromuscular junction and cause MG [6,33]. The absence of thymic myoid cells in thymoma could also provide an explanation for the lack of immunologic tolerance against muscle antigens in patients with thymoma, which results in the development of muscle-directed autoimmunity [6,33].

## 3. Immune Checkpoint Inhibitors for Advanced, Unresectable, or Recurrent TETs

### 3.1. Immune Checkpoint Inhibitor Monotherapy

The indications for use of ICIs targeting the inhibitory immune checkpoints PD-1/PD-L1 and cytotoxic T lymphocyte-associated protein 4 (CTLA-4) have grown exponentially over the past few years, and these drugs, especially antibodies targeting the PD-1/PD-L1 axis, are currently used for the treatment of a wide variety of cancers [38].

Initial evaluation of PD-1/PD-L1-directed ICIs in patients with relapsed TETs was based on the frequent expression of PD-L1 in thymic cancers [21]. Several studies have since been reported, largely in patients with recurrent thymic carcinoma, and reveal a unique pattern of clinical activity and immune-mediated toxicity (Table 1).

Single-agent PD-1/PD-L1-directed immunotherapy is generally associated with response rates of less than 25% and a median PFS of 4 to 15 months in patients with previously treated TETs. A subset of patients derive durable clinical benefit, and longer survival is observed in patients achieving an objective response as compared to those with stable disease [39].

In patients with thymic carcinoma treated with the anti-PD-1 antibody pembrolizumab, high tumor cell PD-L1 expression is associated with a higher response rate and longer survival [40,44]. However, there appears to be no correlation between TMB and response [45].

Analysis of genomic and immunological characteristics of response in a small cohort of patients with thymic carcinoma treated with pembrolizumab showed recurrent mutations in *CYLD*, *CDKN2A*, and *TET2* among responders, whereas *BAP1* and *TP53* were recurrently mutated among non-responders [45]. Responders also had a higher proportion of activated dendritic cells (DCs) and CD4+ memory resting T cells, whereas an increased proportion of M2 macrophages was detected among non-responders [45].

The value of PD-L1 expression as a predictor of response is less clear in patients with thymoma treated with ICIs. Preliminary results from a pilot study of avelumab, an anti-PD-L1 antibody, in 23 patients with relapsed TETs, which included 12 patients with thymoma, showed high pretreatment soluble PD-L1 levels in all patients compared with healthy donors [43]. However, responders had lower B cells and plasmacytoid DCs in their peripheral blood at baseline compared with non-responders [43]. These results are consistent with observations from a previous phase I dose-escalation trial of avelumab, where among seven patients with relapsed thymoma, responders had lower pretreatment B cells along with lower baseline frequencies of Tregs, conventional DCs, and natural killer (NK) cells and a trend toward a higher degree of T cell receptor (TCR) diversity in peripheral blood mononuclear cells (PBMCs) prior to therapy [46].

Immune checkpoint blockade is associated with an increased risk of immune-related adverse events (irAEs) across the histological spectrum of TETs, with a predilection for the development of muscle-directed or neuromuscular toxicity [47]. Collectively, the incidence of all-grade polymyositis, myocarditis, and MG observed in patients with thymic carcinoma treated with anti-PD-1 therapy ranges from 0 to 20%, 0 to 5%, and 0 to 8%, respectively. Corresponding figures for patients with thymoma treated with either anti-PD-1 (pembrolizumab) or anti-PD-L1 (avelumab) therapy are 0–25%, 17–43%, and 8–14% [40,41,42,43,44]. Despite a relatively high incidence of muscle-related irAEs, patients with TETs, including thymomas, do not appear to be at increased risk for the development of other well-recognized irAEs, such as colitis, pneumonitis, endocrinopathies, and skin toxicities, compared with patients with other solid tumors treated with ICIs [48]. These clinical observations can potentially be explained by previously described defects in immunological tolerance associated with TETs, especially against AChR epitopes and muscle antigens [33].

The identification of biomarkers of immune-mediated toxicity in patients with TETs is an active area of research. No correlation has been found between the degree of tumor PD-L1 expression and the development of severe irAEs in patients treated with pembrolizumab [40]. TCR clones were measured in blood before and after treatment and in tumor tissue and muscle in a patient with thymic carcinoma who developed severe myositis and myocarditis after receiving pembrolizumab. Twenty-six TCR clones were found to have increased in frequency in the blood, of which one was detected in both muscle and tumor [44]. In the avelumab trial, lower baseline B cells and NK-T cells and higher CD4+ T cells were present in patients who developed irAEs [43]. Previous observations from the phase I avelumab trial also revealed a trend toward a higher level of pretreatment TCR diversity in PBMCs among patients with thymoma who subsequently developed irAEs [46].

Since immune-mediated myositis occurs at a disproportionately high frequency in patients with TETs receiving immunotherapy, additional efforts have been made to understand the mechanism of muscle damage and identify predictive biomarkers. In patients with thymoma with no clinical history of autoimmune disease, we have shown that the presence of pre-existing AChR autoantibodies is strongly associated with the development of immune myositis [49]. The utility of AChR antibodies as a potential marker of ICI myotoxicity in TETs has been subsequently confirmed in larger datasets [47]. Furthermore, we have used transcriptomic analyses to identify a distinct type of ICI-associated myositis in patients with thymoma, which is characterized by a dense inflammatory infiltrate in muscle biopsies and activation of the interleukin (IL)-6 and type 2 interferon (IFN) pathways. These findings provide a rationale for the evaluation of IL-6 inhibitors, such as tocilizumab, and Janus kinase/signal transducer and activation of transcription (JAK/STAT) inhibitors for the treatment of ICI-associated myositis in patients with thymoma [50].

### 3.2. ICI-Based Combination Therapies

In an effort to improve upon the clinical activity of ICI monotherapy, various combinatorial strategies have been developed and are approved for selected indications, whereas other combinations are under active investigation [51,52]. The broad goal of combination therapy is to further strengthen the anti-tumor immune response generated by ICIs alone while simultaneously targeting immunosuppressive factors that contribute to primary or acquired resistance to ICIs. Several clinical trials are evaluating ICI-based combinations for the treatment of TETs. In this section, we focus on the treatment of advanced, unresectable TETs with ICI-based combinations.

#### 3.2.1. ICIs with Antiangiogenic Drugs

Stimulators of angiogenesis, such as vascular endothelial growth factor (VEGF), promote immune evasion by inhibiting T cell function and DC maturation and contribute to the development of an immunosuppressive tumor microenvironment (TME) by increasing intratumoral Tregs and myeloid-derived suppressor cells (MDSCs) [53].

Combinations of ICIs with antiangiogenic drugs have shown synergistic anti-tumor activity in clinical trials, and an increasing number of such combinations have received Food and Drug Administration approval in recent years [54]. The clinical activity and safety of ICI–antiangiogenic combination therapy for recurrent TETs are under active investigation (Table 2).

The phase II CAVEATT trial evaluated avelumab in combination with axitinib, a potent and selective inhibitor of VEGF receptor tyrosine kinases, in patients with recurrent WHO type B3 thymoma or thymic carcinoma [55]. Notable exclusion criteria included serum antibodies to AChR, titin, or muscle-specific tyrosine kinase and previous treatment with ICIs. In the subgroup of patients who had not received anti-angiogenic therapy previously, the ORR was 47% with a median PFS of 10 months, which compares favorably with the results of ICI monotherapy in a similar patient population. However, clinical benefit was limited in patients who had previously received anti-angiogenic treatment (ORR 15%, median PFS of 4.5 months).

Treatment was well tolerated, and no new safety signals were observed. New-onset irAEs were observed in four (12%) patients, including polymyositis in two (7.4%) patients with thymic carcinoma and one (20%) patient with WHO type B3 thymoma. Clinical activity was observed independent of PD-L1 expression. Genomic characteristics associated with response included mutations in *PBMR1*, *TP53*, and *PI3KCA*.

A recently reported phase I/II trial of nivolumab (an anti-PD-1 antibody) in combination with the VEGF tyrosine kinase inhibitor vorolanib in patients with refractory thoracic cancers included 11 patients with thymic carcinoma (9 patients were enrolled in the dose-expansion cohort). Although only one (11%) objective response was observed in the thymic cohort, patients with thymic carcinoma had the highest disease control rate (66.7%) and the most durable clinical benefit (a median PFS of 9.1 months) among all cohorts.

These results show early evidence of the benefit of ICIs in combination with anti-angiogenic therapy in patients with TETs, especially in individuals who have not received angiogenesis inhibitors previously. Ongoing trials of ICIs with anti-angiogenic drugs will help further clarify the role of these combinations in patients with TETs (Table 3).

#### 3.2.2. Dual Immune Checkpoint Blockade

Preclinical and clinical studies have shown enhancement of an anti-tumor immune response with dual immune checkpoint blockade. Simultaneous targeting of CTLA-4 and PD-1 has been shown to increase CD8+ and CD4+ T cell infiltration into tumors and reduce the activity of immunosuppressive Tregs and MDSCs [57]. Ipilimumab, a recombinant human immunoglobulin (Ig) G1 monoclonal antibody targeting CTLA-4, in combination with nivolumab, is approved for the treatment of several cancers, and various trials are investigating PD-1/PD-L1-directed therapy in combination with drugs targeting other co-inhibitory and co-stimulatory immune checkpoints [52].

The NIVOTHYM study is a two-cohort phase II trial evaluating nivolumab alone or in combination with ipilimumab in patients with advanced WHO type B3 thymoma and thymic carcinoma, with a primary endpoint of PFS at 6 months [42]. Results from the nivolumab plus ipilimumab cohort are awaited. Other trials evaluating dual checkpoint blockade for TETs are outlined in Table 4.

Preliminary results from the KN046 bispecific antibody (anti-PD-L1/anti-CTLA-4) phase II trial (NCT04469725) show an ORR of 16.3% and a median PFS of 5.6 months in 38 evaluable patients. Patients with PD-L1-expressing tumors achieved an ORR of 18.8% and a median PFS of 7.6 months, compared with an ORR of 14.8% and a median PFS of 5.6 months in patients with PD-L1-negative tumors. Grade > 3 treatment-related AEs (TRAEs) were reported in 8 (17.3%) patients [58].

#### 3.2.3. ICIs with Chemotherapy

The ability of cytotoxic chemotherapy to augment the effects of immune checkpoint blockade is well described, and chemoimmunotherapy is now routinely used for the management of several cancers [52,59]. PD-1 inhibitors have been used with platinum-based chemotherapy for the treatment of thymic carcinoma and have been found to be clinically active and safe [60,61,62,63]. A phase II, single-arm trial has evaluated the safety and feasibility of 4–6 cycles of toripalimab, a PD-1 inhibitor, in combination with carboplatin and paclitaxel, followed by maintenance toripalimab until disease progression or the development of intolerable toxicity, as first-line therapy in patients with advanced thymic carcinoma [64]. The primary endpoint was PFS. Fourteen patients were enrolled, and twelve were evaluable for response. The ORR was 41.7%, and grade 3 or 4 TRAEs were observed in five (35.7%) patients, with myelosuppression being the most common grade 3/4 TRAE. These early results support further evaluation of ICIs with chemotherapy for the treatment of TETs. Table 5 lists ongoing trials of chemoimmunotherapy for previously untreated, advanced, or recurrent TETs.

## 4. Immune Checkpoint Inhibitors for Locally Advanced, Resectable TETs

Perioperative immunotherapy is increasingly used for the management of resectable solid tumors [65,66,67,68]. Potential advantages of the early introduction of ICIs include the enhancement of the anti-tumor immune response due to a higher tumor antigen load before resection, a greater chance of downstaging the tumor and increasing the chances of complete resection, early reversal of an immunosuppressed environment, and improved tolerability in treatment-naïve patients [69,70,71]. The clinical benefit of perioperative chemoimmunotherapy in resectable non-small cell lung cancer (NSCLC) has been demonstrated in phase III trials and includes an improvement in pathological response rates and event-free survival (time to disease progression, recurrence, or death). TRAEs are generally manageable, and the majority of patients are able to undergo surgery as planned [72]. Whether similar benefits are seen in patients with resectable TETs without added risks of toxicity or delay in surgery remains to be determined. Results from ongoing clinical trials of chemoimmunotherapy in resectable TETs (Table 6) are eagerly anticipated and should help clarify these issues.

## 5. Immune Checkpoint Inhibitors for Locally Advanced, Unresectable TETs

Locally advanced TETs (TNM stage IIIA–IVA) that are unresectable at diagnosis are treated with platinum-based combination chemotherapy followed by definitive radiotherapy, or with chemoradiotherapy, if clinically indicated [8]. The 10-year risk of relapse in these cases ranges from 29% to 71% in patients with thymoma and 59% to 76% in patients with thymic carcinoma [8]. Consolidation or maintenance therapy after definitive chemotherapy with or without radiation therapy does not currently have a role in the management of these patients.

The immunogenic effects of chemotherapy and radiation therapy are well recognized and include induction of immunogenic cell death (ICD) that generates an immune-infiltrated TME, enhancement of cytotoxic T cell activity, inhibition of immunosuppressive Tregs and MDSCs, increased neoantigen expression, and upregulation of PD-L1 expression [51,73]. An abscopal effect of radiation therapy on non-irradiated metastatic tumors due to the generation of an adaptive immune response has been observed in patients with TETs [74,75,76].

The PACIFIC trial has established the role of the PD-L1 inhibitor durvalumab as consolidation therapy after completion of chemoradiotherapy in patients with unresectable stage III NSCLC and demonstrated a sustained improvement in survival compared with a placebo and no new safety signals in this patient population [77]. In an effort to optimize clinical outcomes, several trials are evaluating ICIs before, with, or after chemoradiotherapy for unresectable stage III NSCLC [78].

Consolidation immunotherapy after chemoradiation has not been evaluated in patients with TETs to date. The adoption of this strategy for locally advanced TETs could be challenging due to a greater propensity for the development of severe immune-mediated toxicity and because of potential differences in clinical activity due to underlying tumor biology. Nevertheless, preclinical and clinical observations of the benefit of consolidation ICI therapy following chemoradiation support the evaluation of this approach in locally advanced, unresectable TETs in carefully designed clinical trials.

## 6. Looking beyond Immune Checkpoint Inhibitors

The vast majority of immunotherapeutic interventions with current clinical applications involve the use of ICIs. However, other forms of immunotherapy at various stages of development include cancer vaccines, immunocytokines and other novel immunomodulators, and cell-based immunotherapeutics, such as adoptive cell therapy (ACT). These interventions are at a nascent stage of development for the treatment of TETs but hold substantial promise for the future.

### 6.1. Cancer Vaccines

Cancer vaccines are designed to generate an immune response against specific tumor antigens in order to elicit tumor regression and improve survival [79,80]. The identification of a suitable TAA is a prerequisite for the successful development of a cancer vaccine.

WT1 is overexpressed in several cancers, including TETs, and serves as an excellent TAA for the development of cancer vaccines [26,81,82]. A peptide vaccine targeting WT1 has been evaluated in 15 patients with TETs in a phase II clinical trial [26]. Treatment was well tolerated, and AEs were generally mild and self-limited. irAEs were observed in two of four patients with thymoma (one case each of late-onset MG and pure red cell aplasia that developed on days 799 and 1015 of treatment, respectively), but not in eleven patients with thymic carcinoma. Although no objective responses were observed, 75% of patients developed disease stabilization, and treatment was associated with generational WT1-specific immune responses. This trial highlights the potential for the development of cancer vaccines for the treatment of TETs.

### 6.2. Cytokine-Based Therapies

Cytokines and chemokines have diverse roles in the TME, which can either support or inhibit tumor growth and the associated immune response [83]. However, until recently, the utility of cytokines as anti-neoplastic therapy has been limited to the use of IL-2 and IFN-α for a limited number of cancers [84]. With the introduction of ICIs and cell-based immunotherapies as effective anti-cancer treatments, there is renewed interest in the use of cytokines or their inhibitors in combination with these drugs to modulate the TME and potentiate the immune response [84]. Cytokine-based treatments are under evaluation for advanced TETs in two clinical trials to date.

#### 6.2.1. Bintrafsup Alfa

Bintrafusp alfa is a first-in-class bifunctional protein consisting of a human IgG1 anti-PD-L1 antibody fused to two transforming growth factor (TGF)-β receptor II molecules that function as a TGF-β trap [85]. When produced excessively and activated by malignant cells, TGF-β promotes tumorigenesis by enhancing the immunosuppressive TME and immune evasion [86]. TGF-β signaling in TECs influences thymopoiesis, and its inhibition impacts the proliferation and cellular composition of the thymic epithelial compartment [87]. In a retrospective analysis of tumor biopsies from patients with TETs, TGF-β expression was detected in 65% of thymic carcinomas and 15% of thymomas. Median OS was shorter in patients with high TGF-β expression (29.5 months vs. 62.9 months; *p* = 0.052) [88]. Bintrafusp alfa is under evaluation in patients with relapsed TETs in a single-arm, phase II trial with a primary endpoint of ORR (Clinical Trial ID: NCT04417660). Key eligibility criteria include no prior treatment with PD-1/PD-L1 ICIs and no significant history of autoimmunity [89].

#### 6.2.2. Nanrilkefusp Alfa (SOT101, Previously SO-C101)

Nanrilkefusp alfa is a fusion protein containing IL-15 and the Sushi+ domain of IL-15 receptor α [90]. IL-15 induces proliferation and cytotoxicity of NK cells and CD8+ T cells without stimulating Tregs [84]. In preclinical models, nanrilkefusp alfa has shown synergy with PD-1 inhibitors and potent anti-tumor activity [91,92]. In a phase I dose escalation study (AURELIO-03; NCT04234113), nanrilkefusp alfa was evaluated as monotherapy and in combination with pembrolizumab in patients with advanced solid tumors [93]. One patient with an ICI-treated recurrent TET received nanrilkefusp alfa monotherapy, resulting in disease stabilization for approximately 20 weeks. No patient with TET received nanrilkefusp alfa in combination with pembrolizumab, and no added toxicity was reported in the combination arm. Although ongoing trials of nanrilkefusp alfa in advanced solid tumors have been reportedly discontinued, preliminary signs of clinical activity support further evaluation of IL-15 agonists in combination with ICIs in patients with advanced TETs.

### 6.3. Targeting Ribosomal Biogenesis

Ribosomes are complex intracellular molecules that play a key role in protein synthesis. There is a growing recognition of the impact of dysfunctional ribosomal biogenesis on tumor growth, metastasis, and resistance to anti-cancer therapy [94]. PT-112 is a novel small-molecule inhibitor of ribosomal biogenesis that causes mitochondrial and endoplasmic reticulum stress in vitro and induces ICD, which releases damage-associated molecular patterns and elicits an anti-cancer immune response [95]. PT-112 is under evaluation in several clinical trials and has previously demonstrated clinical activity in thoracic cancers, including thymoma [96]. In an ongoing phase II clinical trial for recurrent TETs (NCT05104736), ten patients have been treated to date, and eight (89%) out of nine evaluable patients achieved deceased stabilization [97]. The median PFS for thymoma has not been reached, and the median PFS for thymic carcinoma is 6.2 months. TRAEs are generally manageable, and the most common TRAE is peripheral neuropathy (60%). Two patients (20%) experienced a relapse of a previous thymoma-associated autoimmune disease. However, in contrast to ICIs, no new irAEs have been observed. Immune analyses show evidence of early treatment-related immune activation, including an increase in CD8+ T cells, activated CD4+ T cells, and NK cells in peripheral blood, an increase in pro-inflammatory serum analytes, including IFN-γ and tumor necrosis factor-α, and a decrease in immunosuppressive analytes, including VEGF and TGF-β.

### 6.4. Cell-Based Therapies

ICIs have revolutionized cancer care and improved the survival of patients with advanced cancers. However, clinical benefit is still observed in a minority of patients with recurrent tumors, and treatment is complicated by the eventual emergence of acquired resistance. Although rapid advances in unraveling tumor biology have contributed to the development of new drugs and novel strategies to address these issues, there is growing interest in developing personalized cancer therapies that hold the promise of further improving clinical outcomes. ACTs are highly personalized treatments that are designed to elicit an anti-tumor immune response by using either host immune cells that are capable of anti-tumor reactivity or by using manipulated host cells that express anti-tumor T cell receptors (TCRs) or CARs [98]. CAR T cell (CAR-T) therapies have found clinical applications in the treatment of hematological malignancies, but efficacy against solid tumors remains limited due to various reasons, including the low antigenicity of tumor cells, tumor heterogeneity, and the immunosuppressive effects of the TME [99,100]. However, recent advances in TCR- and CAR-based therapies have broadened the scope of these treatments to include solid tumors [100]. Although ACTs are not available for TETs at present, the application of these therapeutic platforms for the treatment of TETs is worthy of consideration in the future.

The identification of tumor-specific antigens is a prerequisite for the successful development of ACT. Attempts are underway to identify novel tumor-specific antigens in thymic carcinomas using genomic and transcriptomic analyses [101]. Early evidence of the utility of this approach for TETs is demonstrated by a complete and durable response to a neoantigen-loaded DC vaccine with neoantigen-reactive T cell adoptive transfer targeting a somatic *CDC73-Q254E* mutation in a patient with recurrent, metastatic thymoma [102].

Examples of more broadly expressed TAAs in TETs that can potentially be used for the development of ACTs include mesothelin and CTAs. Mesothelin is expressed in the majority of thymic carcinomas, and several mesothelin-directed treatments are under development, including CAR-T therapy [28,29]. Gavocabtagene autoleucel, a mesothelin-targeting T cell receptor fusion construct, was evaluated recently in a phase I/II clinical trial (NCT03907852) in patients with selected refractory solid tumors expressing mesothelin (malignant pleural mesothelioma, ovarian cancer, and cholangiocarcinoma) and has shown clinical activity with an ORR of 20% and a 6-month OS rate of 70%. Grade ≥ 3 pneumonitis and cytokine release syndrome were observed in 16% and 25% of patients, respectively [103]. Similar efforts are underway to develop ACTs targeting CTAs, and these treatments can potentially be considered for evaluation in patients with TETs in the future [104,105,106].

## 7. Conclusions

ICIs have altered the landscape of treatment for several cancers and are increasingly being used for the treatment of recurrent thymic carcinomas. The widespread adoption of immunotherapy for TETs is hampered by the risk of developing severe immune toxicity due to defective immune tolerance mechanisms associated with these cancers. As a consequence, immunotherapy is contraindicated for thymomas outside the closely monitored setting of a clinical trial, and caution should be exercised during routine use of ICIs in patients with thymic carcinoma. Identification of biomarkers of response and toxicity of immunotherapy for TETs is an active area of research. As shown by the identification of circulating AChR antibodies as a predictor of immune-mediated muscle damage in patients with TETs, biomarker development has the potential to improve the safety of immunotherapy for patients with TETs and is likely to make the use of these drugs feasible for a larger proportion of patients with thymic cancers. Efforts are also underway to introduce ICIs earlier in the course of the disease. Key considerations for the clinical application of immunotherapy for the management of TETs are summarized in Table 7.

Clinical trials are evaluating the role of immune checkpoint blockade in combination with chemotherapy for the management of resectable TETs and as frontline therapy in treatment-naïve patients with unresectable thymic cancers. If ongoing attempts to identify novel antigens and other immune targets in TETs are successful, it might be possible to broaden the use of immunotherapy beyond ICIs and develop personalized treatment approaches for clinical application.

## Figures and Tables

**Table 1:** Completed clinical trials of immune checkpoint inhibitors for recurrent TETs.

Intervention (Reference)	TET Histology	Endpoints	Number of Evaluable Patients	ORR (%)	Median PFS (mo)	Median OS (mo)	Grade 3 or 4 irAEs (%)	All Grade Muscle or NM irAEs * (%)
*PD-1 inhibitor*								
Pembrolizumab [39]	TC	ORR	40	22.5	4.2	25.5	15.0	7.5
Pembrolizumab [40]	T TC	ORR	7 26	28.6 19.2	6.1 6.1	Not reached 14.5	71.4 15.4	42.9 7.7
Nivolumab [41]	TC	ORR	15	0	3.8	14.1	20.0	20.0
Nivolumab ^^^ [42]	B3T TC	PFS-6	49	12.0	6.0	21.3	57.0	3.7
*PD-L1 inhibitor*								
Avelumab ^†^ [43]	T TC	ORR and safety	12 10	17 20	6.4 14.7	NR NR	58.0 ^‡^ 45.0 ^‡^	25.0 9.0

B3T: WHO B3 thymoma; irAEs: immune-related adverse events; NM: neuromuscular; NR: not reported; ORR: objective response rate; OS: overall survival; PD-1: programmed death-1; PD-L1: programmed death-ligand 1; PFS: progression-free survival; PFS-6: progression-free survival rate at 6 months; T: thymoma; TC: thymic carcinoma; TET: thymic epithelial tumor; * includes polymyositis, myocarditis, myasthenia gravis, or increased creatine phosphokinase; ^^^ results of the single-agent nivolumab cohort from the NIVOTHYM phase II trial of nivolumab alone or with ipilimumab; ^†^ results from the pilot phase of an ongoing phase II trial; ^‡^ all-grade irAE rate.

**Table 2:** Completed clinical trials of immune checkpoint inhibitors with angiogenesis inhibitors for previously treated, recurrent TETs.

Intervention (Reference)	TET Histology	Endpoints	Number of Evaluable Patients	ORR (%)	Median PFS (mo)	Median OS (mo)	All Grade Muscle or NM irAEs * (%)
Avelumab + Axitinib [55].	B3T TC	ORR	32	34	7.5	26.6	9.3
Nivolumab + Vorolanib * [56].	TC	ORR	9	11	9.1	21.0	0

B3T: WHO B3 thymoma; irAEs: immune-related adverse events; NM: neuromuscular; ORR: objective response rate; OS: overall survival; PFS: progression-free survival; TC: thymic carcinoma; TET: thymic epithelial tumor; * phase 2 data.

**Table 3:** Clinical trials of immune checkpoint inhibitors with angiogenesis inhibitors for previously treated, recurrent TETs.

Intervention	Phase	TET Histology	Primary Endpoint	Number of Patients	Clinical Trial Identifier (ClinicalTrials.gov ID)
Pembrolizumab + Lenvatinib	II	B3T or TC	PFS-5	43	NCT04710628
Pembrolizumab + Sunitinib	II	TC	ORR	30	NCT03463460

B3T: WHO B3 thymoma; ORR: objective response rate; PFS-5: progression-free survival rate at 5 months; TC: thymic carcinoma; TET: thymic epithelial tumor.

**Table 4:** Clinical trials of dual immune checkpoint blockade for previously treated TETs.

Intervention	Targets	Phase	TET Histology	Primary Endpoint	Number of Patients	Clinical Trial Identifier (ClinicalTrials.gov ID)
Nivolumab + Ipilimumab	PD-1, CTLA-4	II	B3T or TC	PFS-6	55	NCT03134118
KN046	PD-L1, CTLA-4	II	TC	ORR	66	NCT04469725
KN046 *	PD-L1, CTLA-4	II	TC	ORR	4	NCT04925947
Pembrolizumab + Epacadostat	PD-1, IDO-1	II	TC	ORR	26	NCT02364076

B3T: WHO B3 thymoma; CTLA-4: cytotoxic T lymphocyte antigen 4; IDO-1: indoleamine 2,3-dioxygenase 1; ORR: objective response rate; PD-1: programmed death-1; PD-L1: programmed death-ligand 1; PFS-6: progression-free survival rate at 6 months; TC: thymic carcinoma; TET: thymic epithelial tumor; * after failure of at least one line of immune checkpoint inhibitor monotherapy.

**Table 5:** Chemoimmunotherapy for previously untreated, advanced, or recurrent TETs.

Intervention	Phase	TET Histology	Primary Endpoint	Number of Patients	Clinical Trial Identifier (ClinicalTrials.gov ID)
Carboplatin + Paclitaxel/Nab-Paclitaxel + Pembrolizumab	IV	T or TC	ORR	40	NCT04554524
Carboplatin + Paclitaxel + Pembrolizumab + Lenvatinib	II	TC	ORR	35	NCT05832827

ORR: objective response rate; T: thymoma; TC: thymic carcinoma; TET: thymic epithelial tumor.

**Table 6:** Immunotherapy for resectable TETs.

Intervention	Phase	TET Histology	Primary Endpoint	Number of Patients	Clinical Trial Identifier (ClinicalTrials.gov ID)
Cisplatin + Docetaxel + Pembrolizumab → Surgery → Pembrolizumab consolidation	II	T or TC	mPR	40	NCT03858582
Toripalimab + Chemotherapy *	II	T or TC	mPR, Frequency of SAEs	15	NCT04667793

mPR: major pathologic response; SAEs: severe adverse events; T: thymoma; TC: thymic carcinoma; TET: thymic epithelial tumor; * chemotherapy for thymoma: cisplatin + doxorubicin + cyclophosphamide|chemotherapy for thymic carcinoma: carboplatin + paclitaxel.

**Table 7:** Current clinical application of immunotherapy for the management of thymic epithelial tumors.

**Immunotherapy: Goals and interventions** Immunotherapy broadly aims to eradicate tumor cells through the initiation or amplification of an adaptive immune response by targeting tumor-associated antigens or neoantigens, the modulation of immune checkpoints such as PD-1, PD-L1, or CTLA-4, and the modification of immune cells, including T cells and NK cells. ICIs targeting PD-1, PD-L1, or CTLA-4 are the most common forms of immunotherapy used in clinical practice currently. Other immunotherapeutic interventions in development include cancer vaccines, immunocytokines, cell-based therapies, and other immunomodulatory agents.
**Clinical indications** Advanced or metastatic, unresectable thymic carcinoma after failure of prior platinum-based chemotherapy. Immunotherapy in this setting is currently restricted to the use of the PD-1 antibody pembrolizumab [40,44].
**Immunotherapeutic interventions under investigation** Dual immune checkpoint inhibition [42,58]. ICIs with angiogenesis inhibitors [55,56]. Immunocytokine therapy [89]. Chemoimmunotherapy [64]. Inducers of ICD [97].
**Clinical activity and safety of ICIs** ICI monotherapy for recurrent thymic carcinoma is generally associated with objective response rates of 20–25% and median progression-free survival of 4–6 months. Approximately 10% of patients with thymic carcinoma experience durable clinical benefit [39,40,42,43,44]. Early results from clinical trials evaluating ICI combinations with angiogenesis inhibitors or chemotherapy show higher response rates of 30–40% but require further investigation [55,64]. Patients with TETs are at high risk of severe immune-mediated toxicity, especially muscle and neuromuscular irAEs. The incidence of severe irAEs with ICI monotherapy is 15–20% in patients with thymic carcinoma and 40–70% in patients with thymoma [39,40,41,42,43,44]. Immunotherapy is currently contraindicated outside of a clinical trial for patients with thymoma. Patients with thymic carcinoma receiving immunotherapy should be monitored closely for the development of irAEs. Prompt identification and aggressive management of irAEs are crucial for patients with TETs.
**Biomarkers under investigation** Biomarkers of response and toxicity are not consistently used in routine clinical management of TETs, but biomarker development is an active area of research. PD-L1 expression has shown varying degrees of correlation with clinical response [40,44,55]. Genomic and immunologic determinants of response are under investigation [40,44,45,46,55]. Pretreatment AChR autoantibodies are strongly associated with the development of immune-mediated myositis and myocarditis and are a potential marker of ICI myotoxicity [49]. Baseline evaluation for AChR autoantibodies is recommended prior to the initiation of an ICI. Patients with TETs and detectable AChR autoantibodies should not be treated with ICIs.

AChR: acetylcholine receptor; CTLA-4: cytotoxic T lymphocyte antigen 4; ICD: immunogenic cell death; ICI: immune checkpoint inhibitor; irAE: immune-related adverse event; PD-1: programmed death-1; PD-L1: programmed death-ligand 1; NK: natural killer; TET: thymic epithelial tumor.
